# System-level considerations associated with cow-calf contact in seasonal-calving pasture-based dairy systems

**DOI:** 10.3168/jdsc.2025-0943

**Published:** 2026-02-19

**Authors:** S.J. Hendriks, R.H. Bryant, P. Timmer-Arends, E. Kennedy, J.G. Jago, J.P. Edwards

**Affiliations:** 1 DairyNZ Ltd., Lincoln 7608, New Zealand; 2 Faculty of Agriculture and Life Sciences, Lincoln University, Lincoln 7647, New Zealand; 3 DairyNZ Ltd., Hamilton 3240, New Zealand; 4 Teagasc, Animal and Grassland Research and Innovation Centre, Moorepark, Fermoy, Co. Cork, P61 C996, Ireland

## Abstract

Early separation of cows and calves, a common practice across the global dairy industry, has been regarded as a risk to social license, contributing to growing interest in alternative approaches. Cow-calf contact (CCC) systems, which allow calves to suckle and have contact with the dam after birth, are one such alternative. Since 2020, research undertaken on CCC systems has increased substantially; however, pasture-based dairies remain under-represented in the literature, with only 24% of studies between 1970 and 2025 on calf and cow performance and welfare outcomes being undertaken in pasture systems (n = 24/101). Although dairy industries worldwide have adapted to their unique and often localized constraints, in housed systems, many aspects of the environment can be carefully controlled, allowing management to focus more directly on the animal. In pasture-based systems, cows are managed outdoors, reliant on grazed pasture and commonly use seasonally concentrated (block) calving, where more than 90% of the herd calve within 8 to 10 wk to align calving and the herd's nutritional demands with pasture availability. Feed availability and climatic conditions are more variable, increasing system complexity and interdependence. Consequently, major system-level changes such as CCC in pasture-based contexts require careful consideration with input from relevant stakeholders. Modifications to on-farm practices may create both synergies and trade-offs across the interconnected system, developed over decades to support profitable, sustainable and resilient farming systems. The purpose of this mini review was to synthesize existing knowledge on CCC within block-calving pasture-based dairy systems, identify the specific opportunities and constraints associated with these systems in the context of CCC, and highlight research gaps that need to be considered before its implications can be evaluated.

## Highlights

• Cow-calf contact in pasture-based dairies is less studied than in housed systems.
• Preweaning calf growth is improved, but research on long-term effects is limited.
• Saleable milk yield is reduced, but impacts on cow fertility are understudied.
• Impacts on calf and cow health, behavior, and mental state are largely unexplored.
• Practical, environmental, and economic trade-offs require careful evaluation.

## Graphical Abstract

The graphical abstract illustrates the key outcomes and knowledge gaps surrounding cow-calf contact (CCC) in pasture-based dairy systems. At the center, a cow-calf pair represents performance and welfare outcomes under CCC. Arrows depict main findings: a red downward arrow indicates reduced saleable milk yield, and a green upward arrow shows improved pre-weaning calf growth, consistent with housed studies. Research gaps remain around cow and calf health and cow fertility. At the farm system level, depicted by the landscape below the central image, subheadings highlight broader system factors, such as labor demands, public citizens, economic and environmental sustainability, and farmer wellbeing, that intersect with typical pasture-based attributes such as outdoor management and block-calving (shown in blue). Together, these elements illustrate how CCC interacts with the dynamic relationship between farm systems, stakeholders, and animal performance and welfare outcomes. Images from DairyNZ Inc., New Zealand, with permission.

**Figure:**
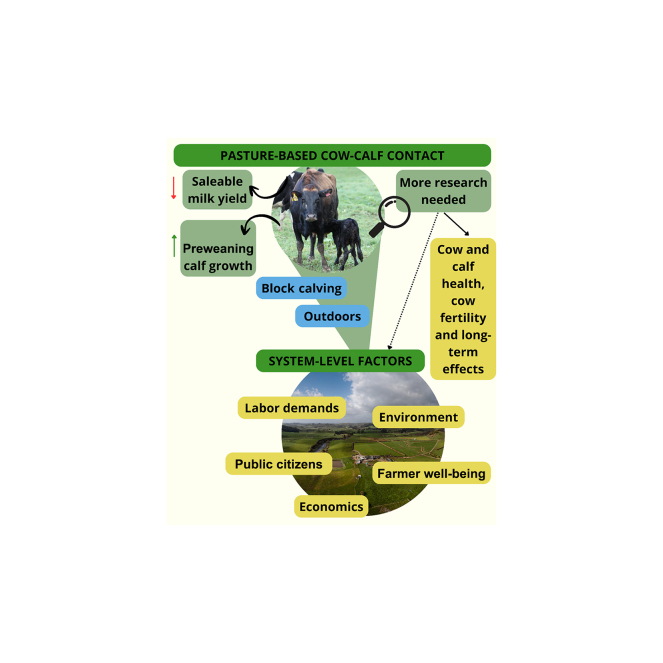

Animal welfare is as a core pillar of sustainable livestock production systems (Keeling, 2025). Across dairy systems, cows and calves are commonly separated within 24 h of birth (von Keyserlingk and Weary, 2007). Public concern over this practice has been examined primarily in countries where cows are housed (Cook and von Keyserlingk, 2024), with comparatively fewer studies exploring citizen perspectives in countries where cows are predominantly kept outdoors (Hötzel et al., 2017; Bolton et al., 2024). However, public support for early separation remains low and is viewed as a possible risk to the long-term sustainability of the dairy industry (Cook and von Keyserlingk, 2024). Dairy industries worldwide have adapted to increasing globalization, shifting policy environments, increasing societal expectations, and environmental constraints, all of which must be managed while maintaining profitability (Hanrahan et al., 2018). Therefore, economic viability alone is not sufficient to ensure sustainable and resilient farm systems.

Pasture-based dairy systems offer welfare benefits including lower disease and injury risk and greater opportunities for cows to express natural behaviors (Mee and Boyle, 2020). However, outdoor management expose animals to variable climatic conditions and associated welfare risks such wet and muddy paddock conditions in winter and spring (Hendriks et al., 2025), whereas block calving concentrates these risks and creates seasonal peaks in labor demand (Deming et al., 2018). Within this context, the separation of cows and calves remains common on-farm practice (Neave et al., 2022), driven by practical considerations including limited shelter for calves born outdoors, long walking distances for milking herds, and the need to maximize pasture utilization (Horan and Roche, 2019), and management decisions intended to support cow and calf health, and colostrum management. But arguments supporting early separation may not align with citizen perspectives (Busch et al., 2017), contributing to growing interest in cow-calf contact (**CCC**) systems (Aytemiz Danyer et al., 2024).

Cow-calf contact is the practice of allowing calves to remain with their dam or a foster (nonmaternal) cow for an extended period after birth, allowing suckling. Although internationally no definition exists of what duration of contact constitutes CCC (Sirovnik et al., 2020), for this review, we included articles where calves remained with their dam or foster cow for more than 24 h after birth. Interest in CCC is increasing, particularly in Europe (Whalin et al., 2025), yet most existing reviews and studies are from housed perspectives (Johnsen et al., 2016; Beaver et al., 2019; Meagher et al., 2019). Perspectives remain limited from intensive pasture-based systems, where management practices, opportunities, and challenges differ markedly from housed systems. Evaluating CCC adoption under pasture-based systems requires consideration not only of cow and calf outcomes, but also system-level trade-offs unique to these systems, as implementation may necessitate changes to management, labor, or infrastructure that could erode current welfare and economic advantages. These system-level factors, in addition to environmental pressures, economic viability, and societal expectations, are areas that are not yet a focus of current pasture-based CCC research. This mini review synthesizes existing knowledge on CCC within intensive, block-calving, pasture-based dairy systems; identifies opportunities and constraints; and highlights research and practice gaps that must be addressed before its implications can be evaluated.

Over 9 in 10 Europeans consider it important to protect the welfare of farmed animals (European Commission, 2023), reflecting an international growing interest in animal welfare. Research on CCC has increased since the early 2020s in both housed and grazing dairy systems (Aytemiz Danyer et al., 2024). Between 2020 and 2024, CCC was the most frequently published calf welfare-related topic in pasture-based dairy research (Verdon et al., 2025), but overall, much of the research to date has been undertaken in Europe and under experimental conditions representative of housed systems (Whalin et al., 2025). In pasture-based systems, CCC research has tended to focus more on calves than cows, but outcomes across both are more evenly represented compared with housed studies (Figure 1). Pasture-based dairies remain under-represented, accounting for only 24% of CCC studies published between 1970 and 2025 (n = 24/101; refer to supplemental material [see Notes]). Research on affective state remains limited across all systems, with studies undertaken exclusively in housed environments (Daros et al., 2014; Neave et al., 2023, 2024b). Consequently, substantial gaps remain in understanding the effects of CCC on performance and welfare outcomes for all stock classes within pasture-based systems. Reflecting the limited evidence base, no reviews have yet distinguished CCC outcomes between housed and pasture-based systems and how differences in management, productivity, and welfare risks may shape the practice of CCC in pasture-based dairy systems.

**Figure 1:**
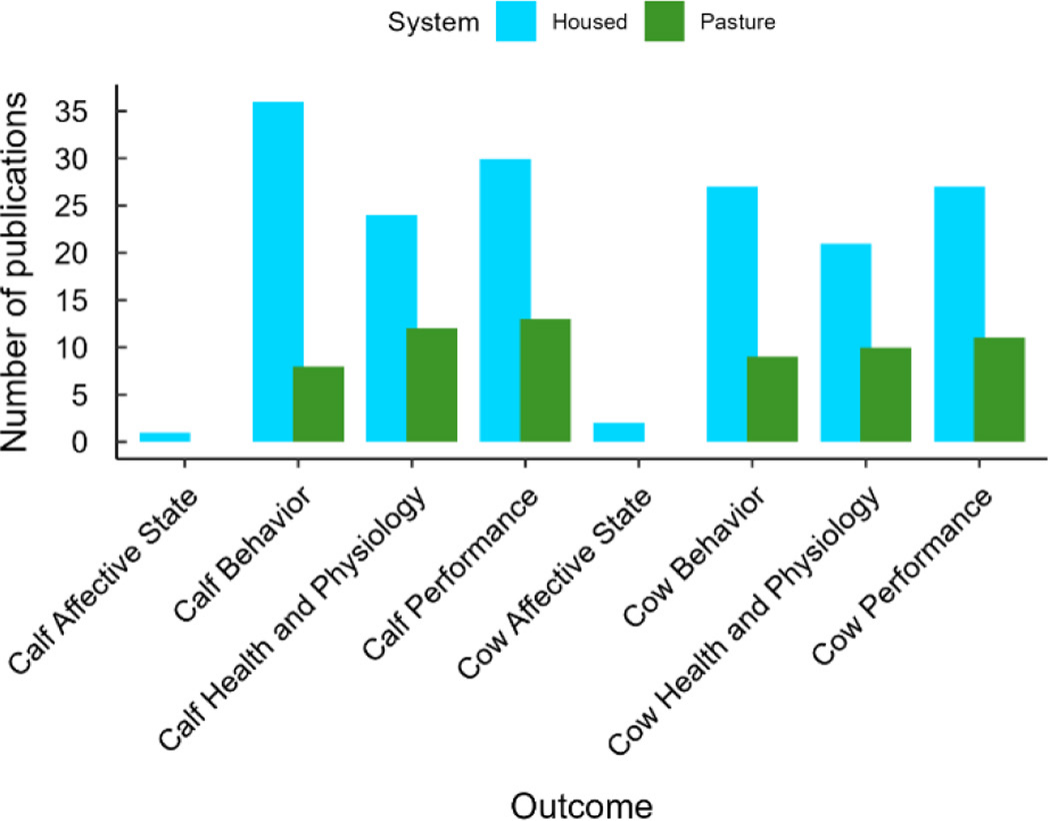
Number of peer-reviewed publications focused on cow-calf contact research relating to health and physiology, behavior, performance, and affective state in calves and cows between 1970 and 2025 undertaken in pasture compared with housed systems. Articles concerning nulliparous heifers were captured under the calf outcomes.

Evidence suggests that improved nutrient provision early in life confers increased first-lactation milk yield (Soberon et al., 2012), primarily through enhanced mammary development (Moallem et al., 2010) and greater liveweight, leading to more energy available for milk production. The benefits of CCC on preweaning calf growth, emphasized in housed systems (Meagher et al., 2019), have also been demonstrated in pasture-based systems (Grøndahl et al., 2007; Mendoza et al., 2010; Ospina Rios et al., 2023; Bryant et al., 2024; Sinnott et al., 2024; McPherson et al., 2025). Elevated preweaning growth in CCC calves is expected due to higher milk consumption (Khan et al., 2022). Importantly, these growth advantages do not necessitate dam rearing (Johnsen et al., 2016). For instance, Bryant et al. (2024) compared full-time CCC to 2 artificially reared groups receiving 6 or 12 L of milk per calf per day (equivalent to 18% and 36% of birth weight, respectively) and reported that both the high-allowance (12 L/d) and CCC groups achieved faster growth and reached weaning liveweight targets earlier than the low-allowance group (6 L/d). In commercial pasture-based systems, it is common to feed calves a daily allowance of ~10% to 15% of birth weight until weaning (Thomson et al., 2018; Schild et al., 2020; Sinnott et al., 2023). Therefore, in systems where early separation remains common practice, achieving preweaning growth equivalent to CCC can be achieved through adjustments in milk allocation.

To date, no evidence exists of carryover advantages between 3 and 9 mo of age in terms of growth rates, liveweights, skeletal development, or future milk yield in pasture-based calves reared under CCC (Bryant et al., 2024; Sinnott et al., 2024). In contrast, housed studies report positive carryover effects in heifers followed to 12 mo (Wagenaar and Langhout, 2007) or the end of their first lactation (Bar-Peled et al., 1997), although the results were often confounded by restricted preweaning milk allowances in artificially reared calves; others report no carryover advantages in calves followed to 4 to 6 mo (de Passillé et al., 2008; Sørby et al., 2024). The limited evidence of carryover effects of CCC in pasture-based systems could reflect a lack of long-term studies (i.e., beyond 9 mo of age) rather than an absence of an effect. Further research is needed to determine whether the preweaning benefits of CCC persist after weaning in both housed and grazing cows, and whether these effects are driven by milk allowance alone or in combination with non-nutritional effects, as this information is essential for evaluating the implications of CCC.

An important focus of CCC systems is whether dam access provides benefits beyond nutrition (Whalin et al., 2025). In pasture-based contexts, the effects of CCC on the transition from milk to solid feed at weaning remain unexplored. Unlike housed calves, which typically transition to TMR and remain indoors, pasture-based calves must develop foraging skills to meet nutritional needs (Costa et al., 2016), adding a dimension to weaning readiness that housed calves experience to a lesser extent. Dam access may support social learning that facilitates this adaptation, but the potential mental distress of separation at weaning could offset benefits if they are transient. By enhancing early-life social and environmental complexity through CCC, same-age, or mixed-age groups (Costa et al., 2016; Field et al., 2023; Miller-Cushon, 2024), calves may benefit from improved integration into novel social groups and faster adaptation to new environments (e.g., onset of grazing when introduced to pasture). However, further research is needed to determine factors that best support weaning readiness in grazing systems. These include rumen development, solid feed intake, postweaning growth, social learning, and longer-term performance, resilience, and competence.

A consequence of CCC is reduced saleable milk yield in high-producing cows (Johnsen et al., 2016; Meagher et al., 2019). Consistent with housed cows (Neave et al., 2024a; van Zyl et al., 2025), lower milk yields during the suckling period are also reported in pasture-based cows (Mendoza et al., 2010; Nicolao et al., 2022; Ospina Rios et al., 2023; Bryant et al., 2024; McPherson et al., 2024). This reduction may be proportionately greater in pasture-based systems, where lower overall milk yields result in a larger proportion of milk being allocated to calves under CCC. Although calf consumption explains part of the milk yield decline, losses typically exceed calf intake and are likely to relate to variation in milk ejection and incomplete milk removal during suckling. For discussions of factors influencing lower-than-expected yields, see Johnsen et al. (2016) and Neave et al. (2024a).

Milking frequency, along with suckling frequency and duration, may contribute to yield differences in pasture-based studies. Unlike most housed studies where machine milk harvesting occurred twice or more daily, in many pasture-based studies, milk harvesting occurred once daily during the suckling period, reporting milk yield losses of ~53% to 57% compared with nonsuckled cows milked twice daily (Bryant et al., 2024; McPherson et al., 2024). More moderate losses (~28% to 35%) have been observed with once-daily milking and part-time contact (Ospina Rios et al., 2023) or twice-daily milking with restricted suckling (Mendoza et al., 2010; Nicolao et al., 2022). Reduced yield lowers cumulative milk production during suckling (Mendoza et al., 2010) and across the lactation (Ospina Rios et al., 2023), representing an economic cost. This cost may be partly offset by the colostrum and whole milk used in artificially reared calves, which would otherwise be nonsaleable, and may be further offset if material longer-term benefits to cow and calf health, calf growth, or system efficiency are realized. Once-daily milking may improve CCC feasibility in pasture systems by reducing labor demands (Sinnott et al., 2024), lowering stress associated with repeated separation for milking (Neave et al., 2024b), and potentially decreasing relative saleable milk losses. Indeed, reduced milking frequencies are already used in grazing herds to manage labor or feed demand (Eastwood et al., 2022), suggesting this approach could be integrated with CCC practices. Nevertheless, the combined effects of milking frequency and suckling on milk yield losses in pasture-based systems require targeted investigation to be fully understood.

After separation and weaning, the effects of CCC on dam milk yield are mixed. Some studies report no differences in housed (Wenker et al., 2022; van Zyl et al., 2025) or grazing systems (Thomas et al., 1981; Mendoza et al., 2010; Ospina Rios et al., 2023), whereas others report persistently reduced yields (housed: Barth, 2020; van Zyl et al., 2025; grazing: Nicolao et al., 2022; Bryant et al., 2024; McPherson et al., 2024). In pasture-based studies, this variation may reflect the carryover effects of, not only impaired milk ejection during suckling, but also differences in feed supply and supplementation, milking frequency during suckling, and the timing and method of separation between studies. Although some of these factors are discussed in housed CCC literature (van Zyl et al., 2025), time required for grazing (7–9 h daily; Neave et al., 2021), feed supply, stage of lactation, and milking frequency are particularly relevant in pasture-based systems. Feed supply is more variable as lactation progresses, with seasonal and regional differences in pasture growth and quality, as well as supplement use and variable prevailing climate conditions, potentially influencing the recovery of milk production following separation (Macdonald et al., 2017). Abrupt separation may further affect time spent grazing if the dam is searching for their offspring. Understanding how these factors, alongside milk ejection, separation method and timing, and suckling frequency, influence postweaning milk yield is essential to evaluate management strategies and assessing the feasibility of CCC in commercial pasture-based systems.

Research on social behaviors under CCC has been undertaken in both calves and cows, although more work has examined maternal behavior and motivation in housed cows (Meagher et al., 2019; Jensen et al., 2024a,b,c) than in grazing cows (Le Neindre, 1989; Ospina Rios et al., 2024). Affiliative interactions between cows and calves under CCC have been described compared with calves artificially reared in groups, with evidence from housed systems indicating that daily contact duration and duration of CCC may affect these behaviors (Whalin et al., 2025). In pasture-based systems, Sinnott et al. (2024) reported greater affiliative social interactions under CCC compared with artificially reared groups, although this may have reflected larger group sizes in the CCC treatments. Although observational in nature, Ospina Rios et al. (2024) reported the expression of maternal and affiliative behaviors within cow-calf pairs. Overall, cows readily express calf-directed behaviors when given access to their calves, challenging the notion that high-producing cows are nonmaternal (Neave et al., 2022). Maternal behavior can vary with temperament; for example, bold cows may be less nurturing (Ospina Rios et al., 2024), although they can still be effective mothers, as calf growth was not impeded by having a bold or less nurturing dam. However, other work does highlight the need for supervision to ensure calves' nutritional needs are met under CCC, as not all calves suckle after birth (Mason et al., 2022).

Another reported benefit of CCC in housed systems is a reduction in abnormal (negative) behaviors in calves, such as cross suckling, non-nutritive suckling, and tongue rolling (Meagher et al., 2019). It remains uncertain whether CCC promotes positive behaviors in calves, such as solitary (object and locomotor) and social play (Whalin et al., 2025). In housed contexts, where calves are reared individually, in pairs, or in groups with varying space availability, comparisons across studies and systems are challenging (Meagher et al., 2019). Individually housed and pair-housed calves have reduced play and increased abnormal behaviors (Roche et al., 2023; Donadio et al., 2025); therefore, when discussing CCC effects in the context of pasture-based systems, where calves are typically reared in groups, we focus on group-housed comparisons (Schild et al., 2020; Sinnott et al., 2023). Many group-housed studies report no differences in solitary (Jensen et al., 1999; Buchli et al., 2017; Waiblinger et al., 2020a) and social play (Jensen et al., 1999; Waiblinger et al., 2020a) or abnormal behaviors (Jensen et al., 1999). Others find increased social play (Wagner et al., 2013) and solitary play (Waiblinger et al., 2020a) and reduced abnormal behaviors in CCC compared with no-contact calves (Fröberg et al., 2008; Roth et al., 2009; Veissier et al., 2013). Evidence from pasture-based systems is limited, with one study reporting no differences in overall play between no-contact and CCC calves, but greater abnormal behaviors in part-time compared with full-time and no-contact calves (Sinnott et al., 2024). Part-time CCC, involving repeated separation and reunion, may elevate stress in calves (Sinnott et al., 2024) and induce negative emotions in dairy cows (Neave et al., 2024b). This is particularly relevant for pasture-based block-calving systems, where part-time or partial contact may be more feasible, especially in large herds. Part-time contact involves periods of physical separation, whereas partial contact restricts nursing using udder nets, nose flaps, or fence-line separation while allowing some contact (Sirovnik et al., 2020). To our knowledge, stress associated with these different contact methods during the suckling period has not been explored in either housed or pasture-based systems; studies to date focus on separation at weaning. Overall, evidence of CCC effects on calf and cow behavior in pasture-based systems remains limited. Future research should assess CCC under conditions typical of pasture-based systems, considering group size, space availability, contact type (i.e., partial, part-time, full-time) (Whalin et al., 2025), and method of separation in part-time contact systems in order to better understand the behavioral benefits and trade-offs of CCC.

Acute behavioral responses to both early separation and prolonged CCC have been documented in cows and calves. Across systems, separation consistently increases vocalizations and activity, irrespective of timing (housed: Flower and Weary, 2001; Veissier et al., 2013; grazing: Mac et al., 2022; Nicolao et al., 2022). Therefore, challenges surrounding weaning and separation remain unresolved. Although these issues are increasingly examined in housed cows (van Zyl et al., 2025; Wegner et al., 2025), ethical questions persist regarding whether stress is better imposed briefly through early separation or over a longer period during (i.e., part-time contact) or following CCC (Whalin et al., 2025). In pasture-based systems, practical considerations around weaning and separation have received little attention, despite their potential importance where calves transition to grazed pasture rather than TMR and weaning occurs later (10–12 wk; Thomson et al., 2018; Schild et al., 2020), compared with housed systems (~8 wk; Cantor et al., 2019). These system-level differences may influence the success of weaning and separation strategies. Addressing these practical and ethical uncertainties in grazing contexts is essential before CCC recommendations can be made.

Calf health outcomes including failure of passive transfer of immunity, respiratory disease, morbidity, mortality, scours, and metabolic state have been examined in CCC calves; however, most evidence comes from housed systems (Beaver et al., 2019) and concerns remain regarding the external validity of these findings due to small sample sizes in housed and pasture-based studies (Whalin et al., 2025). Few pasture-based CCC systems studies have assessed morbidity and mortality, and results are mixed. Although some report no difference in morbidity for CCC compared with artificial rearing (Australia: Ospina Rios et al., 2023; New Zealand: Bryant et al., 2024), others report higher morbidity (Ireland: Sinnott et al., 2024). In the latter study, 25% of calves in the outdoor CCC system were removed due to illness, and 37% required antibiotic treatment, whereas artificial or dam-reared calves housed indoors had better health (6% and 17% antibiotic treatments, respectively) and no removals. Together, these findings align with farmer concerns about increased environmental exposure for calves under variable outdoor conditions (Neave et al., 2022). However, elevated morbidity has only been reported in a single pasture-based study in Ireland, with no comparable findings in the New Zealand or Australia studies, suggesting that local climate, shelter availability, and management practices may influence outcomes. This underscores the importance of replicating studies across diverse regional and system contexts to better understand potential effects of CCC in pasture-based systems (Whalin et al., 2025).

Failure of passive transfer of immunity has been reported under pasture-based CCC (Ospina Rios et al., 2023; Bryant et al., 2024); however, because this occurs within 24 h of birth, it is a risk to both artificial and CCC rearing systems (Cuttance et al., 2017). Therefore, farmers should remain aware of exposure risks and failure of passive transfer of immunity even when calves have dam access, and they should closely monitor calves and provide high-quality colostrum when required. Overall, research investigating calf health under pasture-based CCC remains limited, highlighting the need for further controlled studies. Beyond the calf health issues reported in housed systems, other risks unique to pasture-based CCC, such as parasitism risk in early life, represent an important knowledge gap requiring investigation.

Effects of CCC on cow health and fertility have received little attention in both housed and grazing cows, except for udder health (Beaver et al., 2019). In grazing cows, most studies report no differences in udder health indicators, such as SCC, electrical conductivity, or rapid mastitis test outcomes, between suckled and nonsuckled cows (Fulkerson et al., 1978; Mendoza et al., 2010; Ospina Rios et al., 2023; Bryant et al., 2024; McPherson et al., 2024). Evidence on teat and udder damage is limited: one study found no difference (McPherson et al., 2024), whereas 2 reported greater teat damage under CCC (Thomas et al., 1981; Ospina Rios et al., 2023). No research has investigated potential cumulative effects or changes in udder conformation due to suckling across successive lactations. Similar to udder health, BCS loss during early lactation appears comparable between suckled and nonsuckled cows (Mendoza et al., 2010; Ospina Rios et al., 2023; Bryant et al., 2024; McPherson et al., 2024); however, many studies confound these comparisons by contrasting suckled cows milked once daily with nonsuckled cows milked twice daily, which alters energy demands (Bryant et al., 2024). The effect of CCC on cow physiological health (i.e., blood metabolites, inflammatory markers, minerals, and clinical health score) was examined by McPherson et al. (2025), but no difference was found when compared with nonsuckled cows. Further research is required and should account for milking frequency to more accurately isolate the effects of CCC on cow health and condition.

The effects of CCC on fertility remain poorly understood in housed and grazing systems. Available studies in pasture-based systems report longer calving to first estrus intervals (~3 d) and calving to conception (~30 d), and no difference in calving-to-first-service in suckled compared with nonsuckled cows (Metz, 1987; Mendoza et al., 2010; Bryant et al., 2024). In block-calving systems, where cows must conceive within 8 to 12 wk of the start of breeding and earlier calving maximizes DIM and milk harvested, even modest delays in resumption of estrus or time to conception could have substantial consequences. Fertility and health are key drivers of cow longevity, emphasizing the need for targeted research in pasture-based systems. Current evidence is insufficient to determine CCC effects on health and fertility in both housed and pasture-based systems. Future research should quantify this potential trade-off by examining the risk of key production diseases (e.g., lameness, mastitis), and transition disorders (e.g., hypocalcemia, metritis, retained fetal membranes, clinical ketosis), alongside impacts on fertility and stayability.

Pasture-based CCC studies face similar challenges to housed research regarding the external validity of outcomes (Whalin et al., 2025). Most have been undertaken in controlled conditions with small groups (<25 cow-calf pairs), limiting confidence in extrapolating results to commercial settings. In 2024, herd sizes in Australia and New Zealand averaged 345 and 451 cows, respectively (Dairy Australia, 2025; LIC and DairyNZ, 2025), indicating that the number of preweaning calves present at any time under block calving far exceeds those studied to date, raising uncertainty about the feasibility and outcomes of CCC at scale. Moreover, limited sample sizes in existing studies on health and fertility constrain statistical power and the reliability of conclusions. Large-scale epidemiological and reproductive studies are needed to provide robust evidence; alternatively, meta-analyses could help to synthesize knowledge where large-scale studies are not possible (Whalin et al., 2025). However, without replicated studies under pasture contexts, it remains difficult to undertake such analyses or build scientific consensus to provide evidence-based recommendations for commercial adoption.

Pasture-based dairy systems face growing pressure to remain economically viable while also addressing sustainability challenges. The 4 largest pasture-based geographical regions (New Zealand, Southern Australia, Argentina, and Ireland) supply ~30% of the world's traded dairy products (FAO, 2024), with exports central to several of their economies. These regions represent a substantial proportion of the global dairy herd under grazing conditions. Furthermore, the predominance of block calving and large herds can concentrate resources, risks, and opportunities within distinct periods of the production cycle (Hendriks et al., 2025), meaning welfare outcomes can affect large numbers of animals simultaneously and amplify the consequences of management decisions. Changes to a single component, such as incorporating CCC, must be evaluated in the context of broader system dynamics, as they may exacerbate pressures related to labor, workload, environmental impact, animal welfare, economics, and farmer wellbeing, or alternatively create new opportunities.

The economic implications of adopting CCC under pasture-based systems remain poorly understood and have been cited as a key reason New Zealand farmers are reluctant to adopt such practices (Neave et al., 2022). In housed systems, a stochastic partial budgeting analysis estimated that excluding building costs, CCC adoption reduced short-term farm profitability by ~1% to 5% compared with conventional rearing (Alvåsen et al., 2023), primarily due to reduced saleable milk yield. Although assessing CCC economics solely through milk outputs provides a limited comparison (Meagher et al., 2019), the broader impacts on capital investment, a major driver of farm profitability, remain unexplored.

Infrastructure investment for CCC implementation has been described as modest in housed systems (Whalin et al., 2025), but it could be substantial in pasture-based contexts and represent a major barrier to adoption (Neave et al., 2022). Requirements are likely to vary across hybrid or predominantly outdoor systems, but may include investment in expanded housing or mobile paddock shelter to mitigate welfare risks from environmental exposure (Sinnott et al., 2024). Furthermore, modifications to standard 2-wire fencing used in rotational grazing may be necessary to safely manage cow-calf pairs, further increasing capital costs (McPherson et al., 2024). Although such investment could be recoverable through domestic or niche markets if product premiums are achievable (Whalin et al., 2025), this is less likely in the export-oriented systems typical of pasture-based regions (Roche et al., 2017). To date, there is insufficient evidence quantifying the relative advantages and disadvantages of CCC for cows and calves in pasture-based systems to enable a full economic evaluation. Future analyses should integrate long-term heifer growth and performance, milk yield, calf and cow health, and system-level efficiencies, and potential capital investment requirements to evaluate the feasibility and long-term viability of CCC in these contexts.

A potential benefit of housed CCC is improved job satisfaction, appealing to those motivated by alternative calf rearing approaches (Eriksson et al., 2022). Changes to on-farm labor under CCC systems were identified as a concern by New Zealand farmers (Neave et al., 2022), although only one grazing study has quantified these impacts (Sinnott et al., 2024). Although CCC may reduce some calving-related tasks, such as calf rearing and colostrum collection (Eriksson et al., 2022), the overall workload is likely to shift rather than decrease (Sinnott et al., 2024). New tasks include monitoring suckling and health, managing cow-calf separation and reunions for milking, moving animals and shelters under rotational grazing, and managing weaning and separation. Consequently, CCC is likely to alter labor skill requirements and may raise safety concerns when handling cows with calves at foot, particularly in larger herds (Sinnott et al., 2024). The long-term effects of CCC on dam and calf temperament and responsiveness to humans in large herds are unknown. Although trust-building can foster positive human-animal relationships (Waiblinger et al., 2020b), achieving this at scale may be more challenging. Further research is needed to quantify labor demands under commercial conditions, identify required skills and training, and assess how CCC influences farmer safety and wellbeing and human-animal relationships over time.

Pasture-based systems are likely to be affected by climate change, including increasing temperatures, more frequent extreme weather events, and variable feed supply (Jago et al., 2023). Although no studies have examined CCC under future climate scenarios, implementing CCC may require consideration of potential weather exposure risks for calves under spring-calving, which could necessitate adjustments to calving, altering the synchrony between feed demand relative to pasture growth. Preparing pasture-based CCC for future climates requires careful consideration of exposure risks, management constraints, and future climate impacts.

Although evidence remains limited, CCC may also increase greenhouse gas emissions per kilogram of milk sold due to reduced saleable yield (Mogensen et al., 2022). However, this may be partially offset if improved preweaning growth rates under CCC achieve greater first-lactation milk yields or other long-term benefits. Life cycle assessments tailored to pasture-based dairying are needed to evaluate trade-offs across milk and meat production, resource use, herd performance, and emissions produced.

Although CCC in pasture-based systems may improve growth and reduce abnormal behaviors in calves, as well as promote maternal behavior in cows, evidence remains limited for other performance and welfare outcomes, such as health, longevity, and fertility. The small number of pasture-based studies currently precludes recommending CCC as a best practice for animal welfare. Regions reliant on pasture-based systems require more research to determine whether CCC delivers net welfare benefits for both calves and cows while also accounting for wider challenges, such as labor, economics, infrastructure, and environmental constraints. Consumer perspectives generally favor CCC, but how its perceived value is balanced against their other desired outcomes remains unclear, particularly in pasture-based contexts. Future work should explore how different stakeholders prioritize CCC relative to other welfare, environmental, and labor considerations. A coordinated research agenda should focus on long-term health, performance, and welfare outcomes in cows and calves and address the interdependence among these outcomes and practical feasibility, societal expectation, and sustainability challenges.

## References

[bib1] Alvåsen K., Haskell M.J., Ivemeyer S., Eriksson H., Bicknell K., Fall N., Ahmed H. (2023). Assessing short-term economic consequences of cow–calf contact systems in dairy production using a stochastic partial budgeting approach. Front. Anim. Sci..

[bib2] Aytemiz Danyer I., Diaz Vicuna E., Manfrè C., Contiero B., Forte C., Brscic M. (2024). State of the art of the cow-calf systems in beef and dairy cattle (*Bos taurus*) operations in EU, USA, and Brazil from 1998 to 2023. Res. Vet. Sci..

[bib3] Bar-Peled U., Robinzon B., Maltz E., Tagari H., Folman Y., Bruckental I., Voet H., Gacitua H., Lehrer A.R. (1997). Increased weight gain and effects on production parameters of Holstein heifer calves that were allowed to suckle from birth to six weeks of age. J. Dairy Sci..

[bib4] Barth K. (2020). Effects of suckling on milk yield and milk composition of dairy cows in cow–calf contact systems. J. Dairy Res..

[bib5] Beaver A., Meagher R.K., von Keyserlingk M.A.G., Weary D.M. (2019). Invited review: A systematic review of the effects of early separation on dairy cow and calf health. J. Dairy Sci..

[bib6] Bolton S.E., Vandresen B., von Keyserlingk M.A.G. (2024). “Dear Dairy, It's Not Me, It's You”: Australian public attitudes to dairy expressed through love and breakup letters. Food Ethics.

[bib7] Bryant R.H., Beckett P., Tey L., Burgess R., Curtis J., Heiser A., Turner S.-A., Hodgkinson A.J. (2024). Comparison of suckling and artificial rearing on calf growth and milk requirements in pastoral dairy systems. Anim. Prod. Sci..

[bib8] Buchli C., Raselli A., Bruckmaier R., Hillmann E. (2017). Contact with cows during the young age increases social competence and lowers the cardiac stress reaction in dairy calves. Appl. Anim. Behav. Sci..

[bib9] Busch G., Weary D.M., Spiller A., von Keyserlingk M.A.G. (2017). American and German attitudes towards cow-calf separation on dairy farms. PLoS One.

[bib10] Cantor M.C., Neave H.W., Costa J.H.C. (2019). Current perspectives on the short- and long-term effects of conventional dairy calf raising systems: A comparison with the natural environment. Transl. Anim. Sci..

[bib11] Cook N.B., von Keyserlingk M.A.G. (2024). Perspective: Prolonged cow-calf contact-A dilemma or simply another step in the evolution of the dairy industry?. J. Dairy Sci..

[bib12] Costa J.H.C., Costa W.G., Weary D.M., Machado Filho L.C.P., von Keyserlingk M.A.G. (2016). Dairy heifers benefit from the presence of an experienced companion when learning how to graze. J. Dairy Sci..

[bib13] Cuttance E.L., Mason W.A., Laven R.A., McDermott J., Phyn C.V.C. (2017). Prevalence and calf-level risk factors for failure of passive transfer in dairy calves in New Zealand. N. Z. Vet. J..

[bib14] Dairy Australia (2025). In Focus 2025: The Australian Dairy Industry. https://assets.dairyaustralia.com.au/api/public/content/In-Focus-2025?v=42fe4d04.

[bib15] Daros R.R., Costa J.H.C., von Keyserlingk M.A.G., Hötzel M.J., Weary D.M. (2014). Separation from the dam causes negative judgement bias in dairy calves. PLoS One.

[bib16] de Passillé A.M., Marnet P.-G., Lapierre H., Rushen J. (2008). Effects of twice-daily nursing on milk ejection and milk yield during nursing and milking in dairy cows. J. Dairy Sci..

[bib17] Deming J., Gleeson D., O'Dwyer T., Kinsella J., O'Brien B. (2018). Measuring labor input on pasture-based dairy farms using a smartphone. J. Dairy Sci..

[bib18] Donadio J.P., De-Sousa K.T., Torres R.N.S., Alves T.C., Hötzel M.J., Deniz M. (2025). A meta-analysis approach to evaluate the effects of early group housing on calf performance, health, and behavior during the preweaning period. J. Dairy Sci..

[bib19] Eastwood C.R., Edwards J.P., Bates V. (2022). Science communication and engagement in adaptive farm-systems research: A case study of flexible milking research in New Zealand. Anim. Prod. Sci..

[bib20] Eriksson H., Fall N., Ivemeyer S., Knierim U., Simantke C., Fuerst-Waltl B., Winckler C., Weissensteiner R., Pomiès D., Martin B., Michaud A., Priolo A., Caccamo M., Sakowski T., Stachelek M., Spengler Neff A., Bieber A., Schneider C., Alvåsen K. (2022). Strategies for keeping dairy cows and calves together—A cross-sectional survey study. Animal.

[bib21] European Commission (2023). Attitudes of Europeans towards Animal Welfare. https://europa.eu/eurobarometer/surveys/detail/2996.

[bib22] Field L.A., Hemsworth L.M., Jongman E., Patrick C., Verdon M. (2023). Contact with mature cows and access to pasture during early life shape dairy heifer behaviour at integration into the milking herd. Animals (Basel).

[bib23] FAO (Food and Agriculture Organization of the United Nations) (2024).

[bib24] Flower F.C., Weary D.M. (2001). Effects of early separation on the dairy cow and calf: 2. Separation at 1 day and 2 weeks after birth. Appl. Anim. Behav. Sci..

[bib25] Fröberg S., Gratte E., Svennersten-Sjaunja K., Olsson I., Berg C., Orihuela A., Galina C.S., García B., Lidfors L. (2008). Effect of suckling ‘restricted suckling’ on dairy cows' udder health and milk let-down and their calves' weight gain, feed intake and behaviour. Appl. Anim. Behav. Sci..

[bib26] Fulkerson W.J., Hooley R.D., Findlay J.K. (1978). Improvement in milk production of first calf heifers by multiple suckling. Aust. J. Agric. Res..

[bib27] Grøndahl A.M., Skancke E.M., Mejdell C.M., Jansen J.H. (2007). Growth rate, health and welfare in a dairy herd with natural suckling until 6–8 weeks of age: a case report. Acta Vet. Scand..

[bib28] Hanrahan L., McHugh N., Hennessy T., Moran B., Kearney R., Wallace M., Shalloo L. (2018). Factors associated with profitability in pasture-based systems of milk production. J. Dairy Sci..

[bib29] Hendriks S.J., Saunders K., DeWitt K., Timmer-Arends P., Jago J. (2025). The development of a tool to assess cow quality of life based on system-level attributes across pastoral dairy farms. Animal.

[bib30] Horan B., Roche J.R. (2019). Defining resilience in pasture-based dairy-farm systems in temperate regions. Anim. Prod. Sci..

[bib31] Hötzel M.J., Cardoso C.S., Roslindo A., Von Keyserlingk M.A.G. (2017). Citizens' views on the practices of zero-grazing and cow-calf separation in the dairy industry: Does providing information increase acceptability?. J. Dairy Sci..

[bib32] Jago J., Beukes P., Cuttance E., Dalley D., Edwards J.P., Griffiths W., Saunders K., Shackleton L., Schütz K. (2023). Strategies to minimise the impact of climate change and weather variability on the welfare of dairy cattle in New Zealand and Australia. Anim. Prod. Sci..

[bib33] Jensen E.H., Bateson M., Neave H.W., Rault J.-L., Jensen M.B. (2024). Dairy cows housed both full- and part-time with their calves form strong maternal bonds. Appl. Anim. Behav. Sci..

[bib34] Jensen E.H., Bateson M., Neave H.W., Rault J.-L., Jensen M.B. (2024). Dairy cows' motivation to nurse their calves. Sci. Rep..

[bib35] Jensen E.H., Neave H.W., Bateson M., Jensen M.B. (2024). Maternal behavior of dairy cows and suckling behavior of dairy calves in different cow-calf contact conditions. J. Dairy Sci..

[bib36] Jensen M.B., Munksgaard L., Mogensen L., Krohn C.C. (1999). Effects of housing in different social environments on open-field and social responses of female dairy calves. Acta Agric. Scand. A Anim. Sci..

[bib37] Johnsen J.F., Zipp K.A., Kälber T., de Passillé A.M., Knierim U., Barth K., Mejdell C.M. (2016). Is rearing calves with the dam a feasible option for dairy farms?—Current and future research. Appl. Anim. Behav. Sci..

[bib38] Keeling L.J. (2025). Animal welfare: Part of the solution, not part of the problem in the move toward achieving sustainable development in animal agriculture. Anim. Front..

[bib39] Khan M.A., Heiser A., Maclean P.H., Leath S.R., Lowe K.A., Molenaar A.J. (2022). Growth performance, antibody response, and mammary gland development in New Zealand dairy replacement bovine heifers fed low or high amounts of unpasteurized whole milk. J. Anim. Sci..

[bib40] Le Neindre P. (1989). Influence of cattle rearing conditions and breed on social relationships of mother and young. Appl. Anim. Behav. Sci..

[bib41] LIC (Livestock Improvement Corporation) and DairyNZ (2025). New Zealand Dairy Statistics 2024–25. https://www.dairynz.co.nz/media/oglesqfm/nz-dairy-statistics-24-25.pdf.

[bib42] Mac S.E., Lomax S., Clark C.E. (2022). Behavioral responses to cow and calf separation: Separation at 1 and 100 days after birth. Anim. Biosci..

[bib43] Macdonald K.A., Penno J.W., Lancaster J.A.S., Bryant A.M., Kidd J.M., Roche J.R. (2017). Production and economic responses to intensification of pasture-based dairy production systems. J. Dairy Sci..

[bib44] Mason W.A., Cuttance E.L., Laven R.A. (2022). The transfer of passive immunity in calves born at pasture. J. Dairy Sci..

[bib45] McPherson S.E., Bokkers E.A.M., Sinnott A.M., McFadden M.C., Webb L.E., Kennedy E. (2025). Effect of weaning and cow-calf contact on the physiological and clinical health, performance, and behaviour of dairy cows and their calves. Animal.

[bib46] McPherson S.E., Webb L.E., Murphy J.P., Sinnott A.M., Sugrue K., Bokkers E.A.M., Kennedy E. (2024). A preliminary study on the feasibility of two different cow-calf contact systems in a pasture-based, seasonal calving dairy system: Effects on cow production and health. Animal.

[bib47] Meagher R.K., Beaver A., Weary D.M., von Keyserlingk M.A.G. (2019). Invited review: A systematic review of the effects of prolonged cow-calf contact on behavior, welfare, and productivity. J. Dairy Sci..

[bib48] Mee J.F., Boyle L.A. (2020). Assessing whether dairy cow welfare is “better” in pasture-based than in confinement-based management systems. N. Z. Vet. J..

[bib49] Mendoza A., Cavestany D., Roig G., Ariztia J., Pereira C., La Manna A., Contreras D.A., Galina C.S. (2010). Effect of restricted suckling on milk yield, composition and flow, udder health, and postpartum anoestrus in grazing Holstein cows. Livest. Sci..

[bib50] Metz J. (1987). Productivity aspects of keeping dairy cow and calf together in the post-partum period. Livest. Prod. Sci..

[bib51] Miller-Cushon E.K. (2024). Current research considering social behavior to improve the welfare of commercially raised dairy calves. JDS Commun..

[bib52] Moallem U., Werner D., Lehrer H., Zachut M., Livshitz L., Yakoby S., Shamay A. (2010). Long-term effects of ad libitum whole milk prior to weaning and prepubertal protein supplementation on skeletal growth rate and first-lactation milk production. J. Dairy Sci..

[bib53] Mogensen L., Kudahl A.B., Kristensen T., Bokkers E.A.M., Webb L., Vaarst M., Lehmann J.O. (2022). Environmental impact of dam-calf contact in organic dairy systems: A scenario study. Livest. Sci..

[bib54] Neave H.W., Edwards J.P., Thoday H., Saunders K., Zobel G., Webster J.R. (2021). Do walking distance and time away from the paddock influence daily behaviour patterns and milk yield of grazing dairy cows?. Animals (Basel).

[bib55] Neave H.W., Hvidtfeldt Jensen E., Solarino A., Bak Jensen M. (2024). Exploring factors influencing machine milk yield of dairy cows in cow-calf contact systems: Cow behavior, animal characteristics and milking management. JDS Commun..

[bib56] Neave H.W., Rault J.-L., Bateson M., Hvidtfeldt Jensen E., Bak Jensen M. (2024). Assessing the emotional states of dairy cows housed with or without their calves. J. Dairy Sci..

[bib57] Neave H.W., Rault J.-L., Bateson M., Jensen E.H., Jensen M.B. (2023). Do cows see the forest or the trees? A preliminary investigation of attentional scope as a potential indicator of emotional state in dairy cows housed with their calves. Front. Vet. Sci..

[bib58] Neave H.W., Sumner C.L., Henwood R.J.T., Zobel G., Saunders K., Thoday H., Watson T., Webster J.R. (2022). Dairy farmers' perspectives on providing cow-calf contact in the pasture-based systems of New Zealand. J. Dairy Sci..

[bib59] Nicolao A., Veissier I., Bouchon M., Sturaro E., Martin B., Pomiès D. (2022). Animal performance and stress at weaning when dairy cows suckle their calves for short versus long daily durations. Animal.

[bib60] Ospina Rios S., Lee C., Andrewartha S.J., Verdon M. (2024). Temperament of the dairy cow relates to her maternal behaviour in a pasture-based extended suckling system. Appl. Anim. Behav. Sci..

[bib61] Ospina Rios S.L., Lee C., Andrewartha S.J., Verdon M. (2023). A pilot study on the feasibility of an extended suckling system for pasture-based dairies. Animals (Basel).

[bib62] Roche J.R., Washburn S.P., Berry D.P., Donaghy D.J., Horan B., Beede D.K., Washburn S.P. (2017). Large Dairy Herd Management.

[bib63] Roche S., Renaud D.L., Bauman C.A., Lombard J., Short D., Saraceni J., Kelton D.F. (2023). Calf management and welfare in the Canadian and US dairy industries: Where do we go from here?. J. Dairy Sci..

[bib64] Roth B.A., Barth K., Gygax L., Hillmann E. (2009). Influence of artificial vs. mother-bonded rearing on sucking behaviour, health and weight gain in calves. Appl. Anim. Behav. Sci..

[bib65] Schild C.O., Caffarena R.D., Gil A., Sánchez J., Riet-Correa F., Giannitti F. (2020). A survey of management practices that influence calf welfare and an estimation of the annual calf mortality risk in pastured dairy herds in Uruguay. J. Dairy Sci..

[bib66] Sinnott A.M., Bokkers E.A.M., Murphy J.P., Kennedy E. (2023). A survey of calf housing facilities pre-weaning, management practices and farmer perceptions of calf welfare on Irish dairy farms. Anim. Animals (Basel).

[bib67] Sinnott A.M., Bokkers E.A.M., Murphy J.P., McPherson S., Sugrue K., Kennedy E. (2024). The effects of full-time, part-time and no cow-calf contact on calf health, behaviour, growth and labour in pasture-based dairy systems. Livest. Sci..

[bib68] Sirovnik J., Barth K., de Oliveira D., Ferneborg S., Haskell M.J., Hillmann E., Jensen M.B., Mejdell C.M., Napolitano F., Vaarst M., Verwer C.M., Waiblinger S., Zipp K.A., Johnsen J.F. (2020). Methodological terminology and definitions for research and discussion of cow-calf contact systems. J. Dairy Res..

[bib69] Soberon F., Raffrenato E., Everett R.W., Van Amburgh M.E. (2012). Preweaning milk replacer intake and effects on long-term productivity of dairy calves. J. Dairy Sci..

[bib70] Sørby J., Holmøy I.H., Nødtvedt A., Ferneborg S., Johnsen J.F. (2024). Comparing the effects of contact duration on cow and calf performance beyond separation - A prospective cohort study. Acta Vet. Scand..

[bib71] Thomas G.W., Spiker S.A., Mickan F.J. (1981). Influence of suckling by Friesian cows on milk production and anoestrus. Aust. J. Exp. Agric..

[bib72] Thomson B., Muir P., Ward K. (2018). Calf-rearing systems on dairy farms and specialist calf rearing units: Results of a New Zealand survey. N. Z. J. Anim. Sci. Prod..

[bib73] van Zyl C.L., Eriksson H.K., Bokkers E.A.M., Kemp B., van Knegsel A.T.M., Agenäs S. (2025). Consequences of weaning and separation for feed intake and milking characteristics of dairy cows in a cow-calf contact system. J. Dairy Sci..

[bib74] Veissier I., Caré S., Pomiès D. (2013). Suckling, weaning, and the development of oral behaviours in dairy calves. Appl. Anim. Behav. Sci..

[bib75] Verdon M., Field L., Schütz K., Bryant R. (2025). Invited review: Animal welfare in pasture-based dairy systems—A systematic scoping review to identify progress, priorities, and future directions. J. Dairy Sci..

[bib76] von Keyserlingk M.A.G., Weary D.M. (2007). Maternal behavior in cattle. Horm. Behav..

[bib77] Wagenaar J.P.T.M., Langhout J. (2007). Practical implications of increasing ‘natural living’ through suckling systems in organic dairy calf rearing. NJAS Wagening. J. Life Sci..

[bib78] Wagner K., Barth K., Hillmann E., Palme R., Futschik A., Waiblinger S. (2013). Mother rearing of dairy calves: Reactions to isolation and to confrontation with an unfamiliar conspecific in a new environment. Appl. Anim. Behav. Sci..

[bib79] Waiblinger S., Wagner K., Hillmann E., Barth K. (2020). Play and social behaviour of calves with or without access to their dam and other cows. J. Dairy Res..

[bib80] Waiblinger S., Wagner K., Hillmann E., Barth K. (2020). Short- and long-term effects of rearing dairy calves with contact to their mother on their reactions towards humans. J. Dairy Res..

[bib81] Wegner C.S., Rönnegård L., Agenäs S., Eriksson H.K. (2025). Behavioural responses of dairy cows and calves to fenceline weaning after 4 or 6 months of full cow-calf contact. Animal.

[bib82] Wenker M.L., van Reenen C.G., Bokkers E.A.M., McCrea K., de Oliveira D., Sørheim K.R., Cao Y., Bruckmaier R.M., Gross J.J., Gort G., Verwer C.M. (2022). Comparing gradual debonding strategies after prolonged cow-calf contact: Stress responses, performance, and health of dairy cow and calf. Appl. Anim. Behav. Sci..

[bib83] Whalin L., Barth K., Bertelsen M., Bokkers E.A.M., Ferneborg S., Haskell M.J., Ivemeyer S., Jensen M.B., Johanssen J.R.E., Mejdell C.M., Mughal M., Neave H.W., Vaarst M., van Knegsel A., van Zyl C.L., Wegner C.S., Johnsen J.F. (2025). Invited review: Future directions for cow-calf contact research and sustainable on-farm applications. J. Dairy Sci..

